# The utility of urinary liver-binding fatty acid protein levels as a promising biomarker for early detection of acute kidney injury following coronary angiography: a pre-post study at Suez Canal University Hospital

**DOI:** 10.1186/s12882-025-04681-4

**Published:** 2025-12-24

**Authors:** Mahytab Moustafa Tolba Eladrosy, Mohamed Salah Khedr, Omar Mohamed Saleh, Hanan Hassan Omar, Basma Osman Sultan

**Affiliations:** 1 Department of Internal Medicine, Nephrology Unit, Faculty of Medicine, Suez Canal University, Kilo 4.5, Ring Road, Ismailia, 41522 Egypt; 2 Cardiovascular Medicine Department, Faculty of Medicine, Suez Canal University, Ismailia, Egypt; 3 Clinical Pathology Department, Faculty of Medicine, Suez Canal University, Ismailia, Egypt; 4 Department of Internal Medicine, Faculty of Medicine, Saint Petersburg University, Cairo, Egypt

**Keywords:** Acute kidney injury, Contrast induced nephropathy, Coronary angiography, Contrast media, Liver-Type fatty Acid–Binding protein (L-FABP)

## Abstract

**Background:**

Contrast-induced acute kidney injury (CI-AKI) is a common complication of coronary angiography and percutaneous coronary interventions, with incidence rates ranging from 1% to 25%. Pre-existing renal impairment is the most significant predisposing factor. L-FABP has been evaluated as a biomarker for CIN.

**The aim of this study:**

The aim of this study was early detection of acute kidney injury after coronary angiography by detecting levels of L-FABP as a biomarker to improve the patient’s outcome.

**Methods:**

This is a Pre-post study, as we assessed urinary levels of L-FABP and serum creatinine before and after IV contrast. Sixty-two patients (42 males and 20 females) were recruited from the cardiac catheterization unit of Suez Canal University Hospitals in Ismailia City between December 2021 and January 2022. The study involved medical history, clinical examinations, blood samples for lipid, CBC, creatinine, HbA1C for diabetics, urine L-FABP measurement, and echocardiography. The Mehran score was calculated for each patient to predict risk for CIN and dialysis. Follow-up care for individuals diagnosed with CIN until recovery.

**Results:**

Nearly 70% had hypertension (HTN), 48.4% were diabetic, and 16% had CKD. The mean urinary L-FABP pre-contrast was 187.13 ± 59.07 ng/l, and post-contrast was 201.79 ± 81.8 ng/l. The incidence of CIN was 9.7%. urinary L-FABP levels showed a statistically significant difference within the first 6 h and after 48 h of contrast injection.

**Conclusion:**

In conclusion, CIN is prevalent in advanced-age patients with higher HbA1c levels in patients undergoing coronary interventions. Post-contrast levels showed a significant difference, suggesting potential for early renal injury detection.

## Introduction

Contrast-induced acute kidney injury (CI-AKI) is characterized by the onset of acute kidney injury—defined as either a ≥ 25% rise in serum creatinine from baseline or an absolute increase of 0.5 mg/dL (44 µmol/L)—occurring within 48–72 h after intravascular administration of contrast media (CM), in the absence of another identifiable cause [1].

The true incidence of CI-AKI in the general population remains uncertain, with reported incidence rates varying from around 1% to 25%, influenced by varying definitions, contrast dose and type, administration route, and patient-related risk factors. Impaired baseline renal function remains the strongest predictor, with incidence reaching 20–30% among individuals with pre-existing kidney disease [1].

CI-AKI is linked to increased morbidity and mortality in patients with coronary artery disease. The risk may be due to patient, aging population, concomitant comorbidities, dehydration, and procedure-related factors, such as intra-arterial administration, high osmolar CM, and repeated exposure to contrast [2].

Although the understanding of the complex pathogenesis of contrast induced nephropathy (CIN) is incomplete, the primary model identifies ischemia in the vulnerable outer medullary region of the kidney as pivotal [3].

Following intravascular administration of CM, a prolonged period of renal vasoconstriction occurs due to an imbalance of local vasoactive mediators, such as nitrous oxide, adenosine, endothelin, prostaglandin, and reactive oxygen species (ROS), which are released by the vascular endothelium in direct response to CM cytotoxicity [3].

The combination of cytotoxicity, vasoconstriction, and viscosity in CM agents can induce medullary ischemia/reperfusion injury [3].

Ionic ‘hyper-osmolar’ solutions were initially used but are now rarely administered due to their high nephrotoxicity. Safer agents, such as non-ionic ‘low-osmolar’ or ‘iso-osmolar’ CM solutions, are used for intravascular iodinated contrast administration. The renal injury likely begins immediately after CM administration, with specific biomarkers that could detect it early [4]. 

So much effort has been made in recent years to identify early, specific biomarkers to allow an early diagnosis of AKI and hopefully improve the patients’ outcomes, one of which was fatty acid binding protein (FABP) [5].

The FABP family comprises 15-kDa cytoplasmic proteins found in tissues involved in fatty acid metabolism, where they function as intracellular lipid chaperones that facilitate the transport of fatty acids within the cell. In proximal tubular cells, FABPs carry free fatty acids to mitochondria or peroxisomes for β-oxidation [6].

Two FABP isoforms are expressed in the kidney: liver-type (L-FABP), located in the proximal convoluted and straight tubules, and heart-type (H-FABP), present in the distal tubules. Under normal conditions, L-FABP is undetectable in urine. The L-FABP gene is activated by hypoxic stress, such as those accompanying ischemia–reperfusion injury of the kidney [6].

L-FABP has been evaluated as a biomarker for CIN, with studies showing its best sensitivity and specificity at 4 h post-contrast [5].

Urinary L-FABP levels have been found to increase after 4 h and stay elevated for 48 h in patients with normal S.C who underwent PCI for unstable angina [7].

Urinary L-FABP levels are a useful diagnostic marker for AKI and have prognostic significance. Studies show higher levels in patients with poor outcomes, requiring renal replacement therapy (RRT) [8].

However, studies by Soliman et al. and Hayashi et al. found no correlation between L-FABP levels and CI-AKI occurrence [9]. Additionally, Okumura et al. found that L-FABP cannot predict early AKI [10].

Thus, this study aimed to evaluate whether urinary L-FABP as a biomarker could be used for early detection of AKI post coronary angiography (CA) by measuring its levels before contrast, within the first 6 h following contrast administration, and after 48 h.

## Methodology

This is a Pre-post study as we assessed urinary levels of L-FABP and S.Cr before and after intravenous (IV) contrast administration. A total of 62 patients (42 males and 20 females) were recruited from the cardiac catheterization unit of Suez Canal University Hospitals in Ismailia City between December 2021 and January 2022.

**Inclusion criteria**: All patients scheduled for elective CA during the study period were eligible if they met the following conditions: (i) age ≥ 18 years, (ii) provided informed consent.

**Exclusion criteria**: Patients were excluded if they had any of the following: acute heart failure (HF) or shock, decompensated liver disease, urgent/emergency CA, administration of IV CM within the week before enrollment, history of solid organ transplantation, long-term dialysis, recent exposure to potential nephrotoxic agents or drugs, or concomitant administration of vasoactive drugs.

**Ethical approval** **for this study** was provided by the Ethical Committee at the Faculty of Medicine, Suez Canal University, Egypt. All patients gave their informed consent before their enrollment in this study.

All of the study population were interviewed to obtain history (personal/medical/drugs), clinical examination, and collect blood & urine samples for assessing Lipid profile, Complete blood count (CBC), S. Cr (before receiving contrast and also another sample after receiving contrast 48 h), Haemoglobin (Hb), Haematocrit (HCT), Glycated Haemoglobin (HbA1C) for diabetics, 3 urine samples (before contrast, 6 h & 48 h post-contrast) for measurement of L-FABP by ELISA [11]. A two-dimensional Echocardiography report for each patient was done. Patients received Low-osmolar CM for either CA (25 patients) or percutaneous coronary intervention (PCI) (37 patients).

**Fig. 1:**
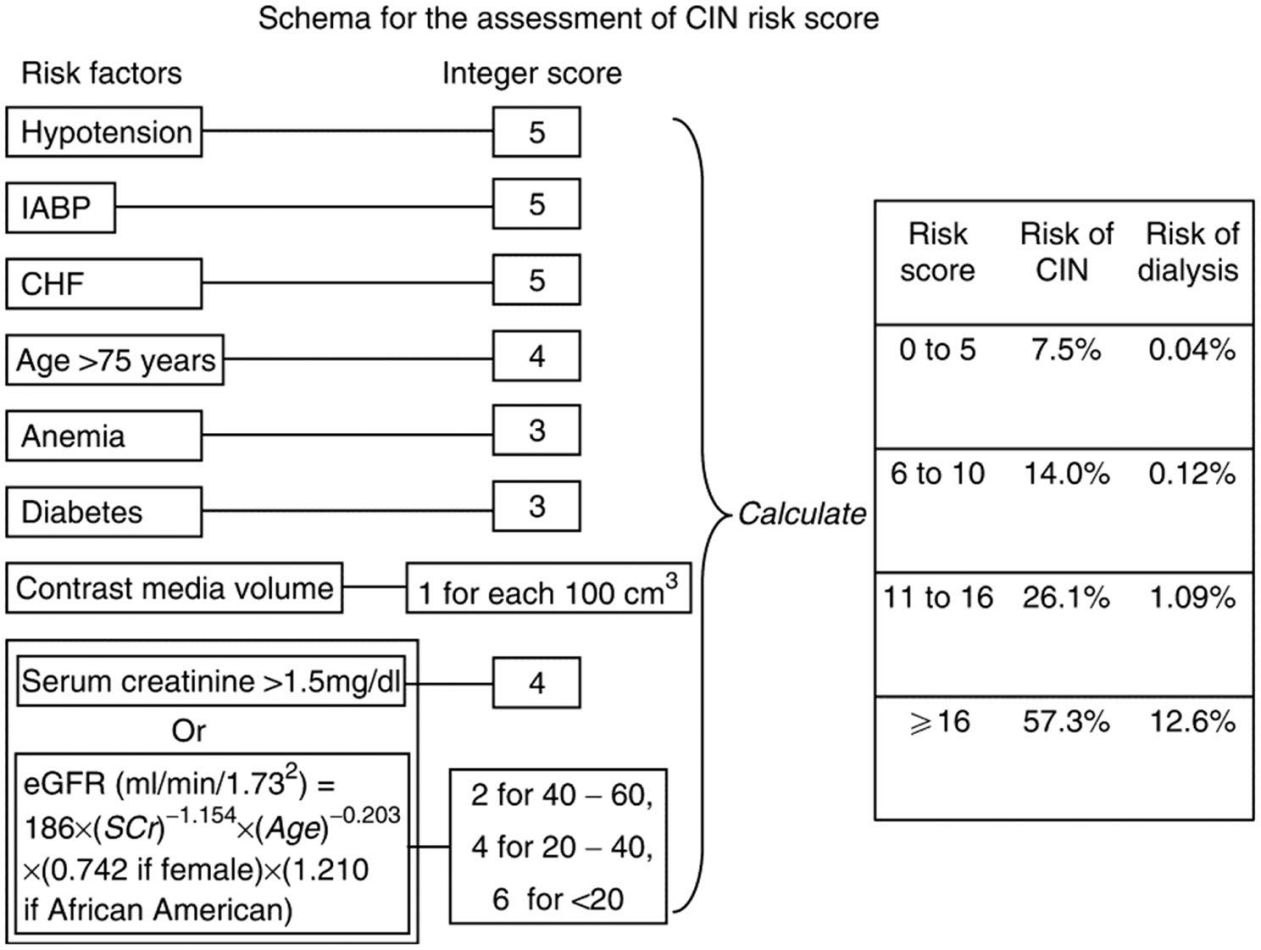
Scheme to define CIN risk score [14]

The Mehran risk score was calculated for each patient. This score is one of the most widely validated and commonly used clinical tools to predict the risk of CIN and subsequent dialysis in patients undergoing CA. It incorporates multiple well-established risk factors, including age, baseline renal function, diabetes mellitus (DM), anemia, hypotension, congestive HF, use of an intra-aortic balloon pump, and contrast volume. By combining these variables, the Mehran score provides a reliable and practical stratification of CIN risk at the bedside. After scoring each risk factor and determining the total score, the predicted risks for CIN and dialysis were derived as shown in Fig. 1 [12, 13]. 

Follow-up care, either inpatient or outpatient, for individuals diagnosed with CIN until they achieve recovery.

## Results

**Fig. 2:**
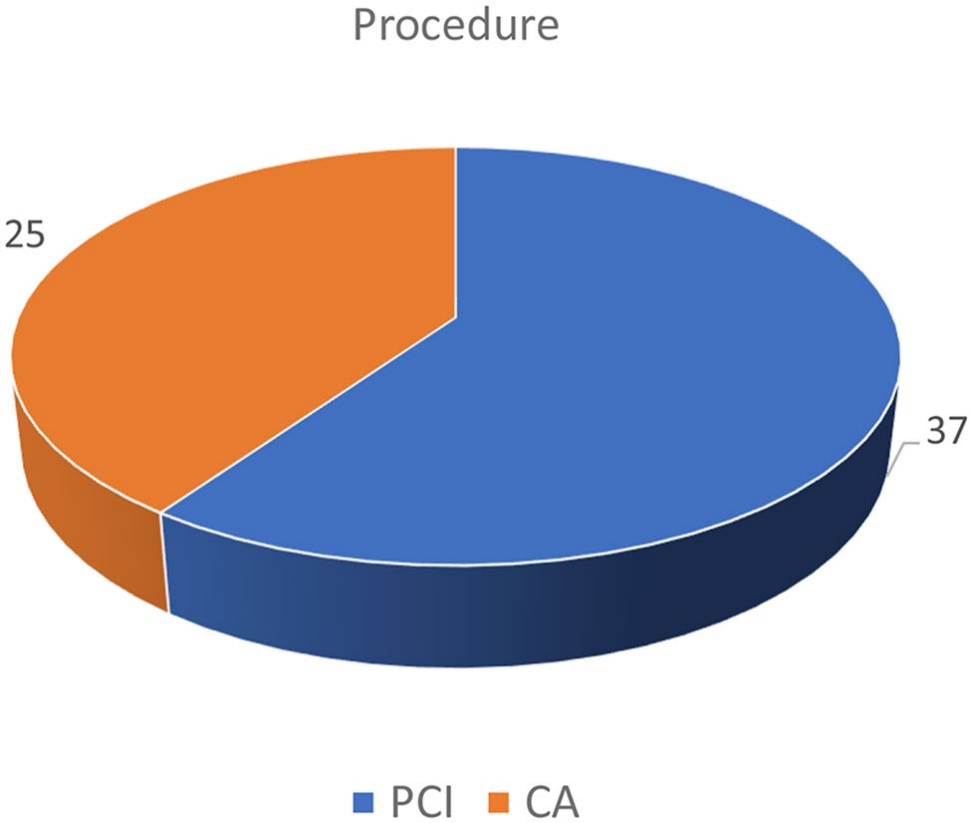
Coronary interventions in the study (*n* = 62)

Table 1 shows the baseline demographic and clinical characteristics of the included patients. The mean age of patients was 58.7 years, ranging from 36 years to 76 years; almost 68% of them were males, 45% of them were non-smokers, and one-third of them were current smokers. Almost 85.5% of them have ischemic heart disease (IHD). 89% of patients presented with chest pain, while the rest (11%) presented with dyspnea. They were scheduled for coronary interventions; 35% of them were due to cardiologist recommendations following previous PCI or CA, 29% were due to current unstable angina pain, and 16.1% were referred from outpatient clinics by stable coronary artery disease. 40% of the patients had a scheduled CA and 60% had a scheduled PCI as demonstrated in Fig. 2. Nearly 70% of patients have HTN, about 48.4% are diabetic (two-thirds of them are on OHD while one-third are on insulin), and 16% of patients have chronic kidney disease (CKD). In Table 2, the mean urinary level of L-FABP pre-contrast was **187.13 ± 59.07** ng/l, while post-contrast within 6 h was **216.5 ± 75.5** ng/l, and after 48 h, **201.79 ± 81.8** ng/l. Pre-contrast renal function evaluation was as follows: Mean S.Cr (0.97 ± 0.25 mg/dl) and mean estimated glomerular filtration rate (e-GFR) (84 ± 21). 83.9% of patients had an e-GFR greater than 60 ml/min/1.73 m2 (stage 1 or 2), whereas 16.1% of the patients were in stage 3. The post-contrast renal assessment was: Mean S.Cr (1 ± 0.34 mg/dl) and mean e-GFR (84.5 ± 25). 83.9% of patients had e-GFR above 60 ml/min/1.73m2 (stage 1 or 2),

11.3% were in stage 3, and 4.8% of them were in stage 4. Around 75% of our patients had a low risk for CIN, 23% had a moderate risk, and 3% had a high risk for CIN. The Incidence of CIN in our study was 9.7%.

Table 3: During the Follow-up of patients after CM administration, the group of patients who developed CIN were older (mean age 67.5 ± 9.7 vs. 57.8 ± 9.3 years, p < 0.05). Traditional risk factors were not significant between the two groups, but mean HbA1c was statistically significant between them (8.4 ± 1.2 vs. 6.9 ± 0.45, respectively, p < 0.0001). It is worth noting that the patients with CIN characteristics are better than those without CIN regarding the pre-contrast kidney function and parameters measured. On the other hand, levels of urinary L-FABP pre-contrast (without statistical significance), while after contrast injection, urinary L-FABP showed a statistically significant difference (p-value 0.001) within the first 6 h and after 48 h of contrast (p-value 0.000).

Table 4: This regression analysis model shows that **age**, **DM**, **baseline pre-contrast GFR**, and **urinary L-FABP levels** were significant independent predictors of CIN in this cohort. Older patients, those with DM, and those with reduced baseline renal function were at higher risk. Importantly, urinary L-FABP emerged as a statistically significant biomarker, supporting its role in the early detection of renal injury following contrast exposure. In contrast, traditional risk factors such as CKD, hypercholesterolemia, anemia, and ejection fraction did not reach statistical significance, possibly due to the small sample size.

**Table 1:** The baseline demographic and clinical characteristics of the studied population

Age (year)	Mean ± SD	58.77 ± 9.74
**Male Gender**	N (%)	42 (67.7%)
**Smoking**	N (%)	20 (32.3%)
**HTN**	N (%)	43 (69.4%)
**DM**	N (%)	30 (48.4%)
**Duration of DM (year):**	Mean ± SD	5.60 ± 8.046
**On insulin**	N (%)	9 (30%)
**On Oral hypoglycemic drugs**	N (%)	21 (70%)
**IHD**	N (%)	53 (85.5%)
**CKD**	N (%)	10 (16.1%)
**Indication:**		
previous PCI	N (%)	22 (35.5%)
Unstable angina	N (%)	18 (29.0%)
Outpatient clinic	N (%)	10 (16.1%)
ECG finding	N (%)	7 (11.3%)
Pre-intervention assessment	N (%)	3 (4.8%)
Dilated cardiomyopathy	N (%)	1 (1.6%)
**Drug History**		
Aspirin	N (%)	54 (87.1%)
Lipid-lowering drugs	N (%)	52 (83.9%)
Beta Blocker	N (%)	47 (75.8%)
ACEI or ARBs	N (%)	37 (59.7%)
Diuretics	N (%)	37 (59.7%)
Oral anticoagulation drug	N (%)	5 (8.1%)
CIN	N (%)	6 (9.7%)
**Mehran score assessment**		
Low risk (≤ 5)	N (%)	46 (74.2%)
Moderate risk (6 to 10)	N (%)	14 (22.6%)
High risk (11 to 15)	N (%)	2 (3.2%)
Very high risk (≥ 15)	N (%)	0 (0%)

**Table 2:** Laboratory findings of the populations studied

Item	Result	
Hb level (gm/dl) Mean ± SD	13.15 ± 1.83	
Hematocrit % Mean ± SD	38.63 ± 4.97	
Hb A1c % Mean ± SD	7.14 ± 0.76	
Positive HCV	7 (11.3%)	
Cholesterol (mg/dl) Mean ± SD	168.56 ± 48.99	
Triglyceride (mg/dl) Mean ± SD	143.73 ± 72.44	
High density lipoprotein (HDL) (mg/dl) Mean ± SD	43.26 ± 12.86	
Low density lipoprotein (LDL) (mg/dl) Mean ± SD	100.48 ± 34.31	
**Pre-contrast Renal Assessment**		
S. Cr (mg/dl) Mean ± SD		0.97 ± 0.25
**e-GFR Mean ± SD**		84.08 ± 21.34
Stage 1 [GFR (90 or above)] N (%)		28 (45.2%)
Stage 2 [GFR (60–89)] N (%)		24 (38.7%)
Stage 3a [GFR (45–59)] N (%)		8 (12.9%)
Stage 3b [GFR (30–44)] N (%)		2 (3.2%)
**Post-contrast Renal Assessment**		
S.cr (mg/dl) Mean ± SD		1 ± 0.34
e-GFR Mean ± SD		84.58 ± 24.68
Stage 1 [GFR (90 or above)] N (%)		32 (51.6%)
Stage 2 [GFR (60–89)] N (%)		20 (32.3%)
Stage 3a [GFR (45–59)] N (%)		6 (9.7%)
Stage 3b [GFR (30–44)] N (%)		1 (1.6%)
Stage 4 [GFR (15–29)] N (%)		3 (4.8%)
**Urinary L-FABP ng/l**		
Pre–contrast Mean ± SD		187.13 ± 59.07
Within 6 h post-contrast Mean ± SD		216.52 ± 75.54
After 48 h post-contrast Mean ± SD		201.79 ± 81.80

**Table 3:** Comparison of characteristics of the patients who developed CI-AKI and those who did not

Variable	CIN (*n* = 6)	Non-CIN (*n* = 56)	*P* value
Age (years) Mean ± SD	67.5 ± 9.7	57.83 ± 9.3	**0.020**
HTN (n, %)	6 (13.9%)	37 (86.1%)	0.099
DM (n, %)	4 (13.3%)	26 (86.7%)	0.305
Duration of DM (years) Mean ± SD	3.83 ± 4.8	5.79 ± 8.3	0.57
IHD (n, %)	6 (11.3%)	47 (88.7%)	0.373
CKD (n, %)	3 (30%)	7 (70%)	**0.048**
Hypercholesterolemia (n, %)	3 (37.5%)	5 (62.5%)	**0.024**
HB A1C Mean ± SD	8.4 ± 1.2	6.9 ± 0.45	**0.000**
Anaemia (N, %)	5 (38.5%)	8 (61.5%)	**0.001**
Ejection Fraction % Mean ± SD	43.7 ± 12	55.3 ± 9.5	**0.007**
Mehran Score Mean ± SD	3.32 ± 2.62	9 ± 3.52	**0.000**
Baseline GFR Mean ± SD	65.3 ± 29.7	86 ± 19.6	**0.022**
Post-contrast GFR Mean ± SD	39.5 ± 18.3	89.4 ± 19.9	**0.000**
Baseline S.cr Mean ± SD	1.12 ± 0.37	0.95 ± 0.26	0.134
Post-contrast S.cr 48 h Mean ± SD	1.72 ± 0.39	0.92 ± 0.23	**0.000**
Contrast volume (ml) Mean ± SD	125 ± 68.9	104.6 ± 55	0.405
Follow up on S.cr after 3 months, Mean ± SD	1.28 ± 0.29	0.97 ± 0.22	0.**013**
Death within 1 year	2 (33.3%)	5 (89.3%)	0.073
L-FABP (ng/mL) levels over time			
0 h (pre-contrast)	228.2 ± 52.5	182.7 ± 58.4	0.073
Within 6 h of post-contrast	312.3 ± 45.8	206.3 ± 70.9	**0.001**
After 48 h of post-contrast	346.2 ± 82	186.3 ± 65.5	**0.000**

**Table 4:** Multivariate logistic regression analysis of risk factors associated with CIN

Item	OR	95% CI		*p*-value
		Upper	Lower	
Age	0.015	0.004	0.027	0.014
CKD	0.023	-0.341	0.387	0.896
Hypercholestrolemia	0.0001	-0.002	0.002	0.906
DM	0.319	0.0154	0.484	0.001
Anemia	-0.008	-0.064	0.048	0.762
EF	-0.009	-0.020	0.002	0.105
Pre-contrast GFR	0.009	0.002	0.016	0.012
L-FAB	0.005	0.001	0.003	0.021

## Discussion

In our study, we assessed the levels of L-FABP in patients who underwent planned CA or PCI and examined the relationship between their levels and the occurrence of CI-AKI.

Sixty-two patients who had either planned CA (40%) or PCI (60%) were included in our study. The average age of the patients was (58.77 ± 9.74) years; nearly 68% of them were men; half of them had DM; 85.5% of the patients had IHD; nearly 70% were known to have HTN; and 16% had CKD. Six (9.7%) of the 62 patients who were recruited developed CI-AKI.

The findings showed that the CIN and non-CIN groups differed statistically significantly in terms of age, CKD, anemia, Hb A1C levels, and hypercholesterolemia. Conventional co-morbidities like DM, its duration, HTN, and IHD did not demonstrate any statistical significance.

Overall, these findings were consistent with those of the **Shuka et al.** study [15], which involved 804 patients and showed that HF, advanced age, and impaired kidney function were the main risk factors for CIN.

A **meta-analysis study** from 2019 showed the predictors of CIN: age, HF, and CKD, which matched our findings. It comprised twelve articles covering 6342 patients in total. However, in contrast to our findings, DM, HTN, and IHD were risk factors for CIN as well [16].

Pre-existing chronic renal disease is one of the strongest patient-related risk factors; in our study, patients with CKD had a higher chance of developing CIN than patients with normal kidney function **(p-value = 0.017).** These outcomes matched those of a recent study by **Çetin et al.** [17]. , which showed that e-GFR was independently associated with the development of CIN. Pre-procedural S.cr levels were found to be independently associated with CIN in **Yuksel and Kose’s study**, which included 925 cardiac patients, and matched our findings [18].

DM is another important risk factor for CIN in many studies [19]. Our study didn’t show any statistically significant difference in either DM or its duration in the two groups. In agreement with **Bağcı et al.‘s** study results of 347 patients, DM occurred at similar rates in both CIN and non-CIN groups [20].

Diabetic patients with CIN had statistically significantly higher HbA1C values. These outcomes were consistent with the findings of the meta-analysis study carried out by **Kewcharoen et al.**, which included eight studies showing that procedural hyperglycemia was linked to an increased risk of CIN even in patients without DM [21].

Regarding procedure-related risk factors, our results showed that CM volume was higher in the CIN group; however, they failed to demonstrate a statistically significant difference between the CIN and non-CIN groups. Evidence to support the finding that CM volume is an independent predictor of CIN was presented by **Yildiz et al.** [22]. Another study by **Saylık et al.** demonstrated that contrast volume/glomerular filtration ratio (Vc/eGFR ratio) was a good predictor for CA-AKI in CKD patients [23].

On the other hand, our results were in agreement with the results obtained by **Özdemir et al.**, who found that the dose of CM was higher in the CIN group without statistically significant differences in both groups [24].

The incidence of CIN in our study was 9.7%, in agreement with a meta-analysis study of 120 studies with 974,898 participants worldwide, the pooled incidence proportion of CIN was 9.06% [25]. Based on the Mehran score, approximately 75% of our patients were at low risk for CIN, 23% were at moderate risk, and 3% were at high risk. According to our study, the Mehran score is a useful predictor of the incidence of CIN with a statistically significant difference **(p-value 0.000)**. These outcomes matched those of **Kumar et al.**, who demonstrated the validity of the Mehran Risk Score for the risk stratification of CIN in cardiac patients [26]. Supporting our observations, the study by **Mirza et al.** employed the Mehran Score to stratify PCI patients for peri-procedural nephroprotective treatment [27].

Regarding levels of urinary L-FABP; urine L-FABP showed a statistically significant difference higher in the CIN group compared to the non-CIN group **(p-value 0.001)** within the first 6 h of contrast and can detect CIN before elevated serum creatinine; additionally, it continued to show a statistically significant difference **(p-value 0.000)** after 48 h of contrast, despite being higher before the contrast without statistically significant difference.

This aligns with **a randomized controlled trial** that identified L-FABP as a useful clinical biomarker for predicting the risk of CI-AKI. The trial also demonstrated that L-FABP enhances practical diagnostic value and contributes to more accurate diagnosis and subsequent management [28].

In line with **Connolly et al.**., another study confirmed the ability of L-FABP to detect early CI-AKI within 4 h following contrast administration. They further highlighted that integrating a Mehran score >10 with 4-hour L-FABP and 6-hour NGAL measurements markedly enhanced diagnostic specificity, reaching 96.7%. It is worth noting that, unlike their study which evaluated serum L-FABP, our assessment was based on urinary L-FABP levels [5]. The same significance was demonstrated by another study by **Manabe et al.**, which found that before contrast medium exposure, urinary L-FABP levels were significantly higher in patients with CI-AKI than in those without CI-AKI [8].

The findings of our study were consistent with the findings of **Lin et al.**‘s study, which involved 591 patients and demonstrated the significance of L-FABP for the early detection of CI-AKI, particularly in patients with CKD [29]. A study by **Soliman et al.** showed that renal function decline in patients with acute on-top CKD following coronary angiography was correlated with urinary L-FABP, which was useful not only for the early diagnosis of CI-AKI but also for prognostic value. Patients with progressive CKD had significantly higher urinary L-FABP at three months as compared to baseline [9].

Another study conducted by **Noirie et al.** revealed that the use of L-FABP measurements in clinical practice can result in improved patient outcomes through faster results and responses, more accurate diagnosis and monitoring, and higher rates of volume expansion and other preventive measures [28].

Urinary L-FABP can predict hospital-acquired AKI in a **meta-analysis study** including 38,725 patients. It demonstrated how well L-FABP/Cr predicted the occurrence of AKI with various etiologies in critically ill patients [30].

However, some studies have been unable to find any significance for the biomarker involved. For example, **Zdziechowska et al.‘s study**, which examined the levels of numerous markers (NGAL, L-FABP, KIM-1, and IL-18), found no significant correlations between the decline in e-GFR and any of the biomarker levels. This study’s small sample size, population of 52 patients, was one of its limitations [2].

Compared with other AKI biomarkers, L-FABP generally surpasses serum creatinine and eGFR in early detection, demonstrates performance comparable to NGAL, shows stronger predictive ability in CKD populations, and correlates well with tubular injury markers such as KIM-1. Although Cystatin-C also offers early detection, its predictive accuracy in CKD is lower, whereas NGAL and the combination of [TIMP-2]•[IGFBP7] have shown substantial promise for early stratification of risk. Despite these advancements, challenges remain, including the absence of methodological standardization, variable availability, and inconsistent predictive performance across studies. As a result, consensus is growing in favor of multi-marker panels incorporating L-FABP, NGAL, KIM-1, and Cys-C may provide superior early diagnosis, prognostic precision, and personalized prevention approaches for CIN [11, 31–33]. 

Another prospective study found that renal resistive index in Doppler was a more accurate predictor of CIN than renal biomarkers, with L-FABP levels being comparable in both groups and failing to demonstrate any significance of L-FABP in the CIN group [34].

## Conclusion

**In conclusion**, this study offers valuable insights into the clinical profile of patients undergoing coronary interventions, with particular emphasis on the incidence and determinants of CIN. A substantial proportion of patients presented with comorbidities such as cardiovascular disease, HTN, DM, and pre-existing kidney disease. The incidence of CIN was 9.7%, with advanced age and elevated HbA1c emerging as significant predictors. Although pre-contrast urinary L-FABP levels did not vary between patients who developed CIN and those who did not, post-contrast levels—particularly within the first 6 h and again at 48 h—showed significant differences, that may suggest potential utility as an early biomarker of renal injury. These observations support the importance of monitoring and targeted risk stratification in individuals undergoing coronary procedures, especially older patients and those with poorly controlled DM.

Further research is warranted to corroborate the predictive performance of urinary L-FABP, including evaluation at additional time points to more accurately determine peak sensitivity and specificity. Comparative analyses with other biomarkers may also enhance its diagnostic and prognostic value. Moreover, larger, multicenter studies are essential to confirm and extend the generalizability of our findings across broader patient populations. Finally, future work should not only aim to validate biomarkers but also evaluate clinical interventions that could be implemented following early CI-AKI detection using L-FABP, with the goal of improving long-term renal and cardiovascular outcomes.

## Data Availability

Datasets generated during and/or analyzed during the current study are available from the corresponding author upon reasonable request. The data are not publicly available because it contains information that could compromise the privacy of research participants.

## Declarations

### Statistical analysis

Data was collected and coded, then analyzed using the Statistical Package for the Social Sciences (SPSS) program version 22.0.0.0. Data was presented as tables and graphs as appropriate. Continuous variables were expressed as mean and standard deviation, while categorical variables were expressed as numbers (N) and percentages (%). Comparisons were performed using the T-test (for quantitative data) and chi-squared-test (χ2), or Fisher’s Exact test was used (for qualitative data). One-way analysis of variance (ANOVA) was used for parametric variables, and the Kruskal-Wallis test was used for non-parametric variables.

### Generative AI and AI-assisted technologies in the writing process

We declare the use of Grammarly as a tool for language editing and improving readability.

## Abbreviations

(H-): Heart-type
(L-): Liver-type
AKI: Acute Kidney Injury
ANOVA: One-way analysis of variance
CA: Coronary Angiography
CBC: Complete Blood Count
CI-AKI: Contrast-induced acute kidney injury
CIN: Contrast-Induced Nephropathy
CKD: Chronic Kidney Disease
CM: Contrast Media
Cys-C: Cystatin-C
DM: Diabetes Mellitus
e-GFR: Estimated glomerular filtration rate
FABP: Fatty acid binding protein
Hb: Haemoglobin
HbA1C: Glycated Haemoglobin
HCT: Haematocrit
IHD: Ischemic heart disease
IL-18: Interleukin-18
IV: Intravenous
KIM-1: Kidney Injury Molecule 1
L-FABP: Liver Fatty acid-binding protein
N: Numbers
NGAL: Neutrophil gelatinase-associated lipocalin
PCI: Percutaneous coronary intervention
ROS: Reactive oxygen species
RRT: Renal replacement therapy
S. Cr: Serum creatinine
SPSS: Statistical Package for the Social Sciences
